# Supplementary material for: Targeting Glutamine Transporters as a Novel Drug Therapy for Synovial Sarcoma
Source: Cancers (Basel). 2025 Dec 19;18(1):15. doi: 10.3390/cancers18010015 (PMC12784809; doi:10.3390/cancers18010015)
Supplement: Supplementary file 1 [file cancers-18-00015-s001.zip › 251215 Supplementary Data.pdf]

Supplementary Data (Figures)

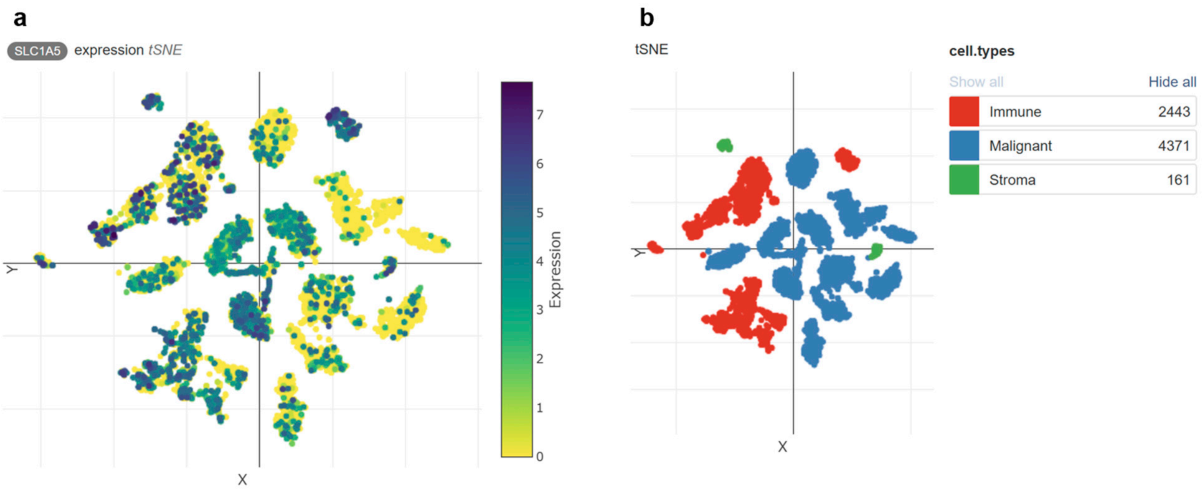

Figure S1. Single-cell RNA sequencing analysis of synovial sarcoma cell populations and SLC1A5 expression [21]. (a) t-SNE plot showing clustering of three primary cell types: malignant, immune, and stromal. (b) t-SNE plot showing the expression level of SLC1A5 across cell clusters.

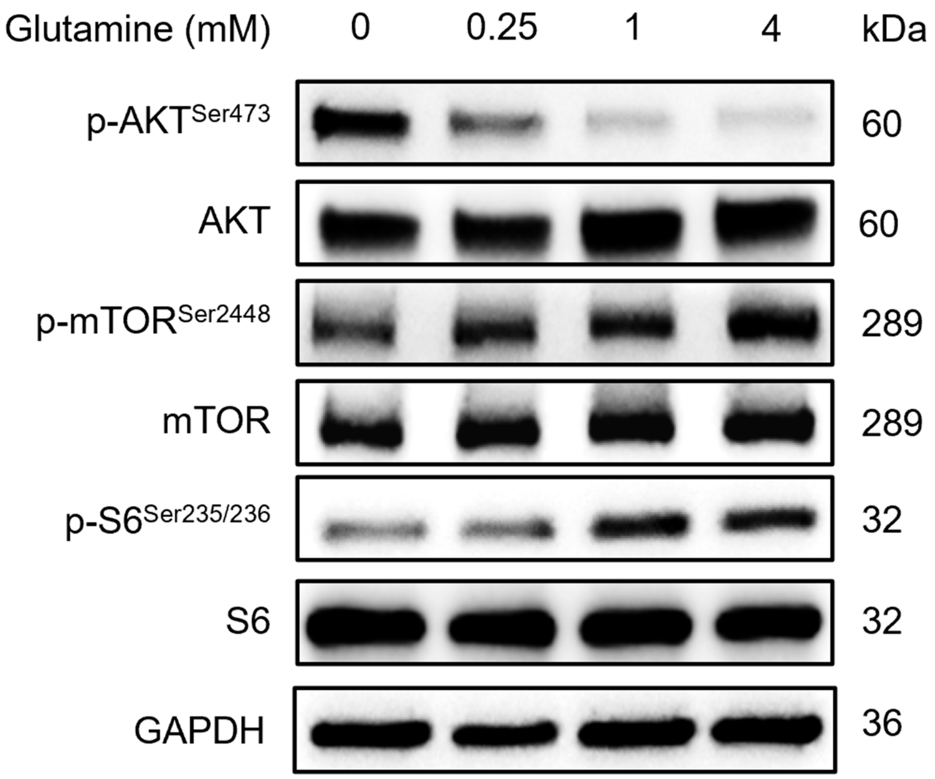

Figure S2. Western blot analysis of key proteins in the AKT/mTOR pathway after treating HS-SY-II cells with varying glutamine concentrations for 24 h. GAPDH was used as a loading control.

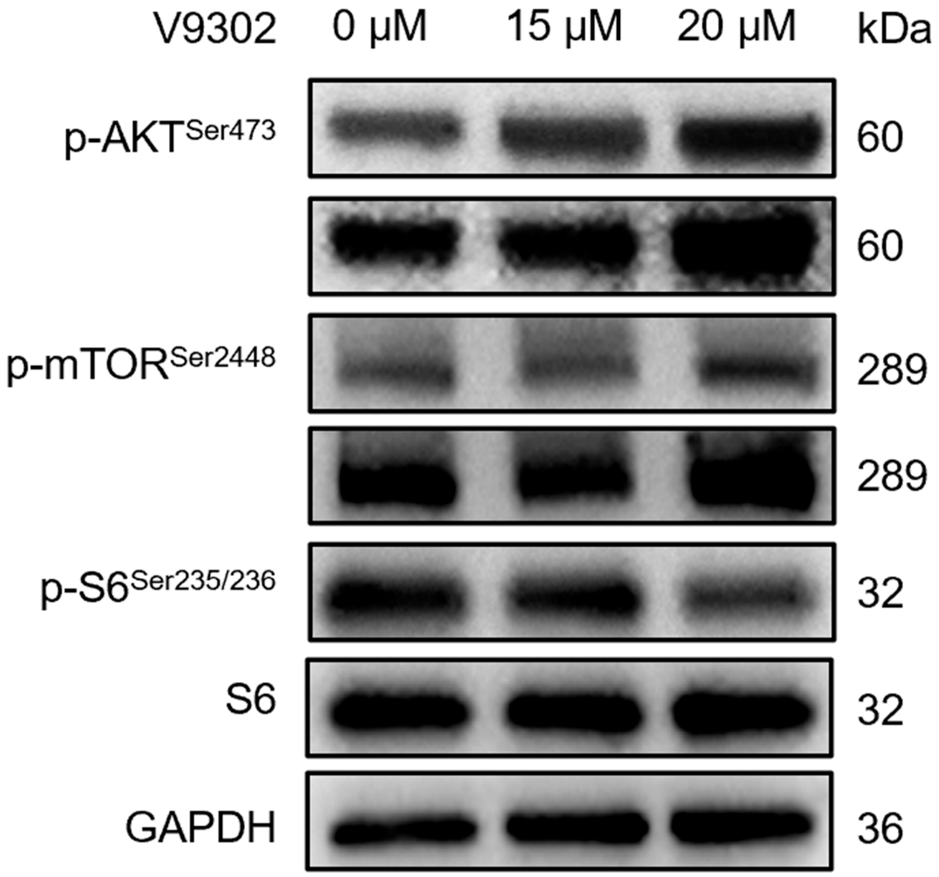

Figure S3. Western blot analysis of key proteins in the AKT/mTOR pathway after treating HEK293 cells with different V9302 concentrations for 24 h. GAPDH was used as a loading control.
