# Supplementary figures and images for: Targeting Glutamine Transporters as a Novel Drug Therapy for Synovial Sarcoma
Source: Cancers (Basel). 2025 Dec 19;18(1):15. doi: 10.3390/cancers18010015 (PMC12784809; doi:10.3390/cancers18010015)

**Figure 1c.**

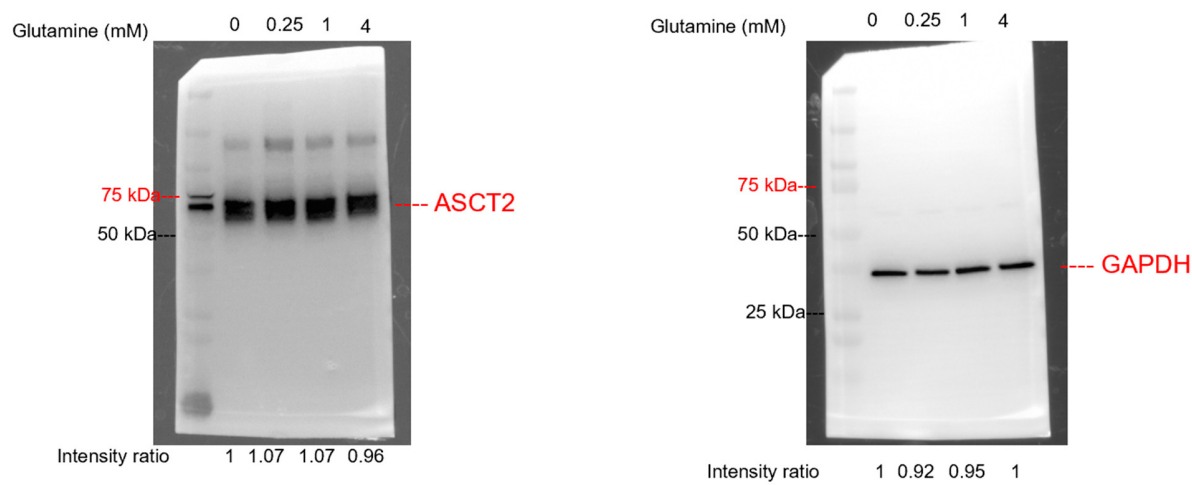

Figure 4a

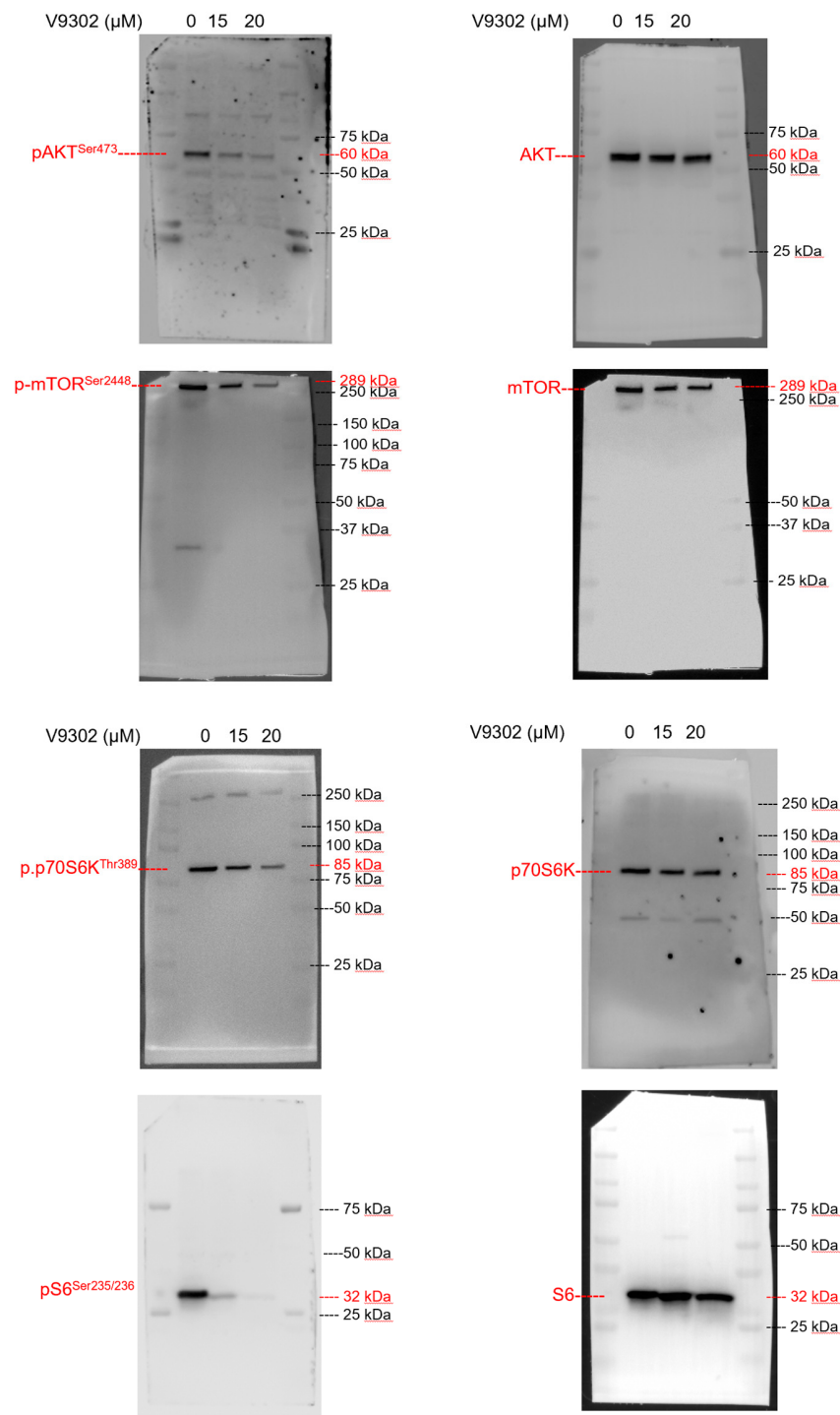

**Figure 5c.**

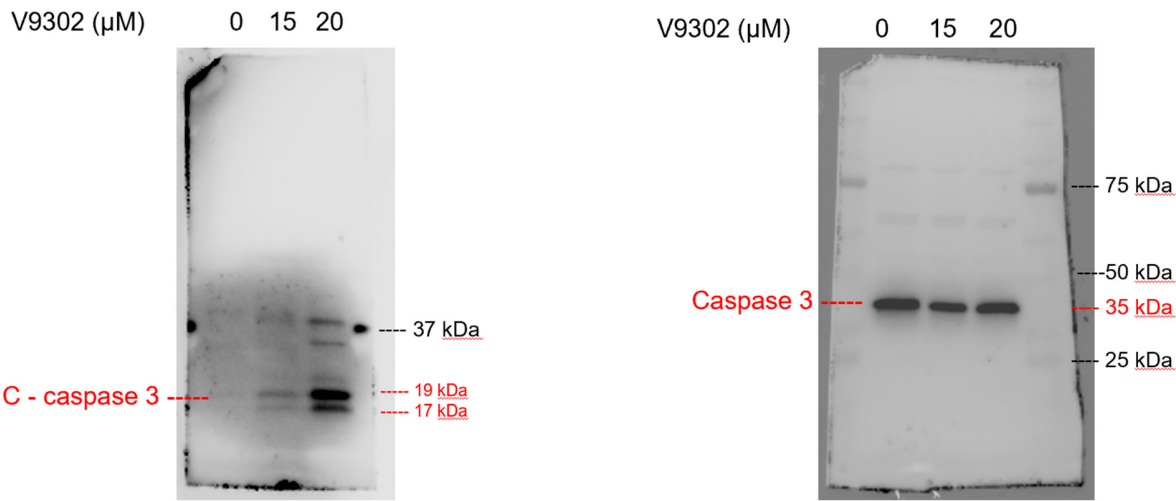

Figure S2.

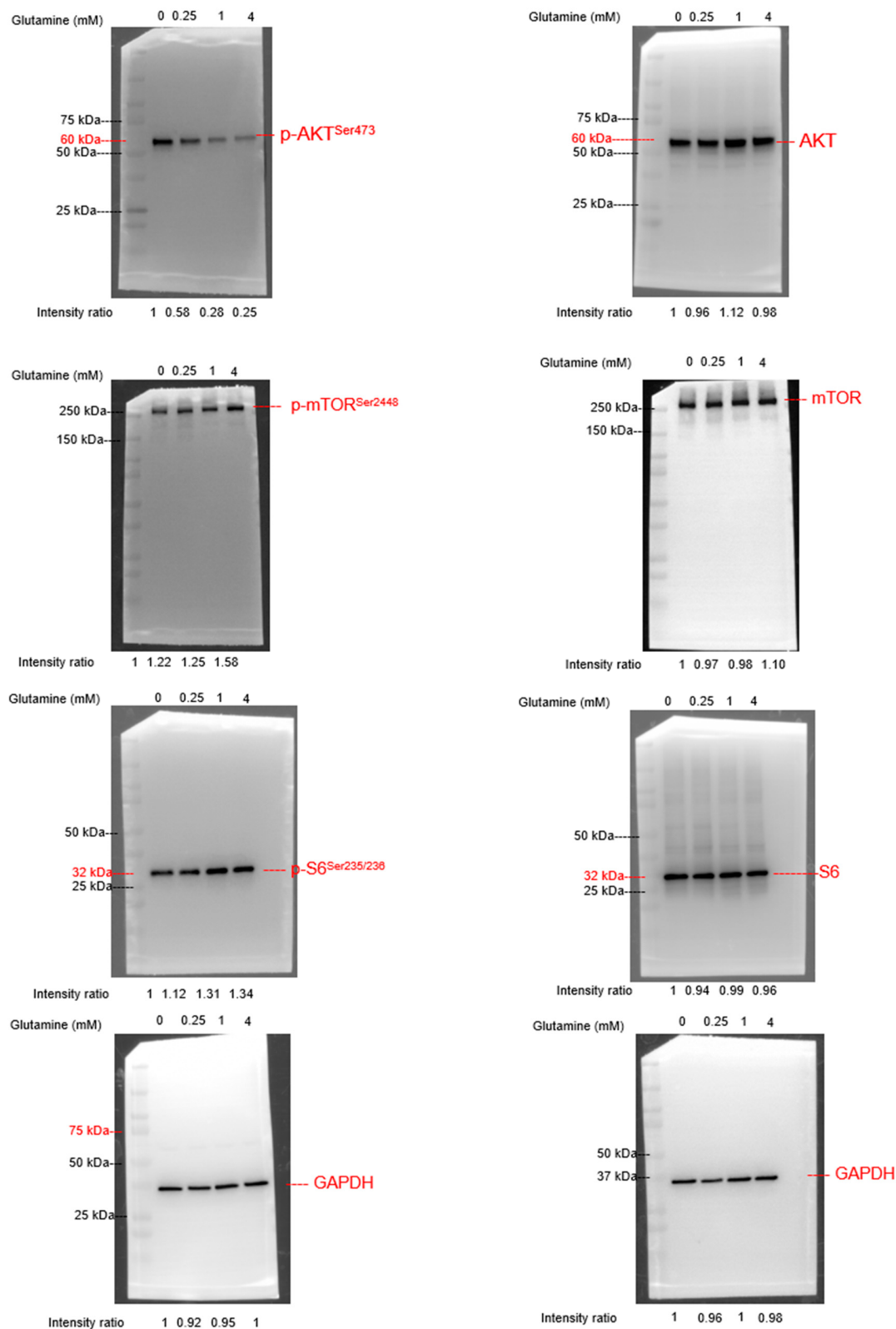

**Figure S3.**

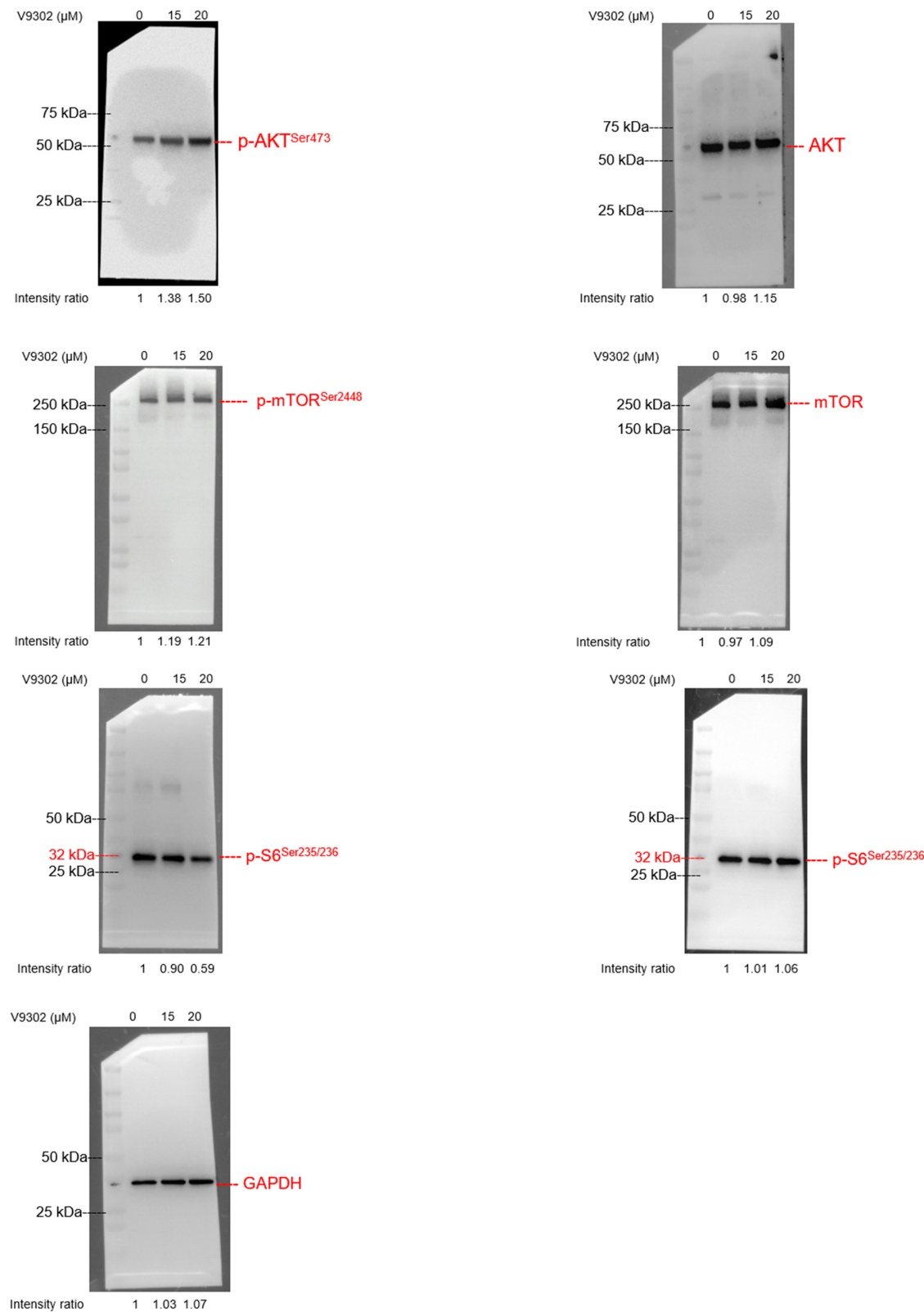

Supplement: Supplementary file 1 [file cancers-18-00015-s001.zip › 251215 Supplementary Materials-The uncropped bolts of Western blots.pdf]
